# Arterial and Venous Thromboembolism in *ALK*-Rearrangement-Positive Non-small Cell Lung Cancer: A Population-Based Cohort Study

**DOI:** 10.1093/oncolo/oyad061

**Published:** 2023-04-04

**Authors:** Oded Icht, Avi Leader, Erez Batat, Lilach Yosef, Tzippy Shochat, Daniel A Goldstein, Elizabeth Dudnik, Galia Spectre, Pia Raanani, Ariel Hammerman, Alona Zer

**Affiliations:** Institute of Oncology, Davidoff Cancer Center, Rabin Medical Center, Petah Tikva, Israel; Sackler School of Medicine, Tel Aviv University, Tel Aviv, Israel; Sackler School of Medicine, Tel Aviv University, Tel Aviv, Israel; Institute of Hematology, Davidoff Cancer Center, Rabin Medical Center, Petah Tikva, Israel; The Community Medical Services Division, Clalit Health Services, Tel Aviv, Israel; Institute of Oncology, Davidoff Cancer Center, Rabin Medical Center, Petah Tikva, Israel; Statistical consulting unit, Rabin Medical Center, Petah Tikva, Israel; Institute of Oncology, Davidoff Cancer Center, Rabin Medical Center, Petah Tikva, Israel; Sackler School of Medicine, Tel Aviv University, Tel Aviv, Israel; The Community Medical Services Division, Clalit Health Services, Tel Aviv, Israel; Lung Cancer Service, Assuta Medical Centers, Tel Aviv, Israel; Ben-Gurion University of the Negev, Beer-Sheva, Israel; Sackler School of Medicine, Tel Aviv University, Tel Aviv, Israel; Institute of Hematology, Davidoff Cancer Center, Rabin Medical Center, Petah Tikva, Israel; Sackler School of Medicine, Tel Aviv University, Tel Aviv, Israel; Institute of Hematology, Davidoff Cancer Center, Rabin Medical Center, Petah Tikva, Israel; The Community Medical Services Division, Clalit Health Services, Tel Aviv, Israel; Sackler School of Medicine, Tel Aviv University, Tel Aviv, Israel; Oncology Division, Rambam Health Care Campus, Haifa, Israel

**Keywords:** anaplastic lymphoma kinase (*ALK*), NSCLC, thrombosis, venous thromboembolism, arterial thromboembolism

## Abstract

**Introduction:**

There is scarce data regarding the incidence of venous thromboembolism (VTE) and arterial thromboembolism (ATE) in the molecular subtypes of non-small cell lung cancer (NSCLC). We aimed to investigate the association between *Anaplastic Lymphoma Kinase (ALK)*-positive NSCLC and thromboembolic events.

**Methods:**

A retrospective population-based cohort study of the Clalit Health Services database, included patients with NSCLC diagnosed between 2012 and 2019. Patients exposed to ALK-tyrosine-kinase inhibitors (TKIs) were defined as *ALK*-positive. The outcome was VTE (at any site) or ATE (stroke or myocardial infarction) 6 months prior to the diagnosis of cancer, until 5 years post-diagnosis. The cumulative incidence of VTE and ATE and hazard-ratios (HR) with 95% CIs were calculated (at 6- 12- 24 and 60-months), using death as a competing risk. Cox proportional hazards multivariate regression was performed, with the Fine and Gray correction for competing risks.

**Results:**

The study included 4762 patients, of which 155 (3.2%) were *ALK*-positive. The overall 5-year VTE incidence was 15.7% (95% CI, 14.7-16.6%). *ALK*-positive patients had a higher VTE risk compared to *ALK*-negative patients (HR 1.87 [95% CI, 1.31-2.68]) and a 12-month VTE incidence of 17.7% (13.9-22.7%) compared to 9.9% (9.1-10.9%) in *ALK*-negative patients. The overall 5-year ATE incidence was 7.6% [6.8-8.6%]. *ALK* positivity was not associated with ATE incidence (HR 1.24 [0.62-2.47]).

**Conclusions:**

In this study, we observed a higher VTE risk, but not ATE risk, in patients with *ALK-*rearranged NSCLC relative to those without ALK rearrangement. Prospective studies are warranted to evaluate thromboprophylaxis in *ALK*-positive NSCLC.

## Teaser

There is scarce data regarding the incidence of venous thromboembolism and arterial thromboembolism in the molecular subtypes of non-small cell lung cancer (NSCLC). This study investigated the association between ALK-positive NSCLC and thromboembolic events.

**Implications for Practice**

There is scarce data regarding the incidence of venous and arterial thromboembolic events (VTE and ATE, respectively) in the genetic subtypes of non-small cell lung cancer (NSCLC). This retrospective, population-based cohort study demonstrated that *ALK*-positive patients had 87% higher risk of developing VTE and that the ATE rates were comparable between *ALK*-positive and *ALK*-negative patients. In *ALK*-negative patients, both VTE and ATE were associated with increased 5-year all-cause mortality, but *ALK*-positive patients did not demonstrate a such association. These results suggest that the genetic profile of a tumor should be incorporated into clinical scores that aid in thromboprophylaxis decision-making.

## Introduction

Solid malignancies are associated with an increased risk for venous thromboembolism (VTE)^1-3^ and arterial thromboembolism (ATE).^4-6^ While clinical scoring systems such as the Khorana score have been developed to identify patients with cancer at increased risk for VTE and can guide thromboprophylaxis, their performance in specific malignancies, especially lung cancer, is suboptimal.^7-10^ Given the implications and poor outcomes of patients with cancer-associated VTE and ATE,^11-13^ more accurate prediction tools are needed for patients with lung cancer, to select patients who may be candidates for thromboprophylaxis. Tumor-specific markers, such as somatic genomic alterations, may help improve VTE risk stratification, as exemplified in a recent study.^14^

In non-small cell lung cancer (NSCLC), advances in molecular testing in the last decade have introduced specific genomic subtypes with variable clinical behavior. *Anaplastic Lymphoma Kinase* (*ALK)* rearranged NSCLC is one such subtype identified in 5% of all patients with pulmonary adenocarcinoma, and usually affects younger, non-smoking patients.^15^ Recent retrospective studies, including from our group, have shown a 3- to 5-fold higher VTE rate in *ALK-positive* NSCLC.^16-18^ This is also the case in *ROS1* rearranged NSCLC representing 1%-2% of the NSCLC population.^19^ Data on ATE incidence in specific molecular subtypes are scarce. Limitations of these studies include small sample size, selection bias of patients undergoing genomic sequencing, and mostly single-centre settings.

We aimed to address these knowledge gaps and investigate the association between *ALK*-positive NSCLC and VTE/ATE in a large nationwide cohort of patients with advanced lung cancer treated in tertiary and community-based centers.

## Methods

### Design and Patient Sample

A retrospective population-based cohort study was performed using the Clalit Health Services (CHS) database. CHS is both a healthcare payer and provider, covering over half of the Israeli population, with individual patient data recorded in a centralized electronic database. Accordingly, the CHS database has a sample that is fairly representative of the Israeli population.^20^ The study included patients diagnosed with advanced lung cancer between January 2012 and June 2019, defined as both of the following: International Classification of Diseases-9th revision (ICD-9) codes compatible with a diagnosis of pulmonary malignancy (162.X) and treatment with systemic anti-cancer therapy. No exclusion criteria were defined.

Patients were indexed on the day of lung cancer diagnosis. Records were followed from 6 months prior to index date, and until 5 years post index, or until death, whichever occurred first. The follow-up for outcomes began 6 months prior to the index date since outcomes occurring in this time-period can be considered to be cancer-associated.^21^

### Variables and Measurements

Patient data documented at study index included the following: age, sex, smoking status (current/non-smoker), body mass index (BMI), platelet count, white blood cell count, hemoglobin level, Charlson’s comorbidity index,^22^*ALK*—inhibitor treatment. The Khorana score at the index was calculated according to data availability.^7^

### Groups and Exposure

The study exposure was the presence of *ALK* rearrangement. *ALK*-positive NSCLC was defined as ≥2 *ALK*-inhibitor prescriptions (crizotinib or alectinib) at any time during follow-up since these medications require pre-authorization in Israel, and were the only *ALK*-inhibitors reimbursed and utilized in Israel as first-line therapy during the study period. NSCLC patients with 0-1 *ALK*-inhibitor prescriptions were classified as *ALK*-negative.

### Outcomes

The outcomes of interest included a first diagnosis of VTE or ATE during the follow-up period. ATE was defined as primary ICD-9 diagnosis of myocardial infarction (MI) or stroke. VTE was defined using ICD-9 codes for deep vein thrombosis (DVT) at any site or pulmonary embolism (PE) (ICD-9 codes are detailed in Supplementary Table S1).

### Statistical Analyses

Patient characteristics are presented as median (inter-quartile range [IQR]) for continuous variables and as frequency for categorical variables. Baseline characteristics were compared between patients with or without outcome events during follow-up, using *t*-test for continuous normal variables, Wilcoxon for skewed variables, and Fisher`s exact test for categorical variables. All analyses were performed separately for VTE and ATE.

The cumulative incidence of outcomes throughout follow-up, with corresponding 95% CIs, was calculated for the whole cohort and also separately for *ALK*-positive and *ALK*-negative subgroups. Death (not due to VTE or ATE) was considered as a competing risk using the Fine and Gray estimator.

A Cox proportional hazards model was used to calculate the hazard ratios (HR) and their corresponding 95% CI for outcome events (VTE or ATE) between *ALK*-positive and *ALK*-negative groups, with death as a competing risk (Fine and Gray method). Two separate multivariate cox proportional hazards regression models (one for VTE and the other for ATE) were used to calculate associations between the following baseline variables and VTE/ATE: *ALK* status (positive/negative), age (continuous), sex (male/female), smoker (past or current/none), Charlson comorbidity index (continuous), Khorana score (continuous). Death was considered a competing risk in this analysis.

Statistical analysis was performed using the SAS Vs 9.4 software (SAS Institute, North Carolina, USA).

## Results

### Patient Characteristics

A total of 4762 patients met the inclusion criteria for this study. Patient characteristics are detailed in Table 1. The majority of patients (63%) were male, 72% of patients had documented history of past/current smoking. Seventeen patients (0.35%) had only one *ALK* tyrosine kinase inhibitor (TKIs) prescription and were not defined as *ALK*-positive, while 155 (3.2%) had ≥2 *ALK*-inhibitor prescriptions and were defined as *ALK-*positive. The median follow-up time was 15.2 months (IQR 7-29.61). During the follow-up period, 45.16% of patients in the *ALK*-positive group, and 70.26% of patients in the *ALK*-negative group died. The VTE and ATE incidence in all patients is detailed in the Supplemental Results and Supplementary Fig. S1.

**Table 1.** Characteristics of the participants at baseline, stratified for VTE/ATE status during follow-up*.

Variable (at NSCLC diagnosis)		All, *n* (%)	VTE^*^, *n* (%)	No VTE^*^, *n* (%)	ATE^*^, *n* (%)	No ATE^*^, *n* (%)
All patients, *n* (% of all)		4762 (100)	673 (14)	4089 (86)	311	4451
Age, median (range)		67 (21-98)	65 (21-94)	67 (26-98)	68 (39-93)	67 (21-98)
Sex	Male	3017 (63)	387 (57)	2630 (65)	204 (66)	2813 (63)
	Female	1731 (36)	282 (43)	1449 (35)	106 (34)	1625 (37)
*ALK* rearranged^‡^		155 (3.2)	45 (6)	110 (3)	12 (4)	143 (3)
BMI	<35	4328 (91)	617 (92)	3711 (91)	276 (89)	4052 (91)
	≥35	257 (5)	28 (4)	229 (5)	19 (6)	238 (5)
	Missing	177 (4)	28 (4)	149 (4)	16 (5)	161 (4)
Smoker^§^		3447 (72)	440 (65)	3007 (74)	217 (70)	3230 (73)
Charlson comorbidity index, mean (range) [*n* = 4542]		2.50 (0-20)	2.29 (0-12)	2.53 (0-20)	2.51 (0-20)	2.50 (0-18)
Khorana score, mean (range) [*n* = 3513]		1.5 (1-5)	1.4 (1-4)	1.5 (1-5)		

^*^VTE/ATE at any point from 6 months prior study index to 5 years post-index. Index date defined as date of NSCLC diagnosis.

^‡^Defined as ≥2 *ALK*-inhibitor prescriptions (crizotinib or alectinib).

^§^Past or current smoking.

Abbreviations: *ALK*, anaplastic lymphoma kinase; ATE, arterial thromboembolism; BMI, body mass index; NSCLC, non-small cell lung cancer; VTE, venous thromboembolism

### VTE According to *ALK* Status

**Figure 1.**
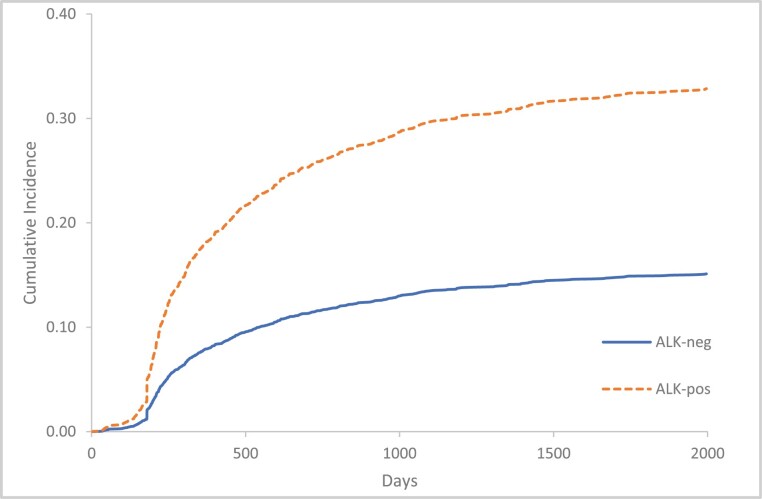
Cumulative incidence of VTE, according to *ALK* rearrangement status. Outcome events counted from 6 months prior to study index to 5 years post-index. Index date defined as date of NSCLC diagnosis. Death considered as a competing risk. *ALK*, anaplastic lymphoma kinase; VTE, venous thromboembolism.

During the follow-up period, 45/155 (29%) patients with *ALK*-positive NSCLC experienced a VTE compared to 628/4607 (13%) in *ALK*-negative patients. The cumulative incidence of VTE among patients with *ALK*-positive NSCLC (95% CI) at index (representing the prior 6 months) and 6-, 12-, 24-, and 60-months post-index were 5% (3.7%-6.6%), 17.7% (13.9%-22.7%), 22.6% (17.7%-29.7%), 27.5% (21.2%-35.7%), and 32.8% (26.3%-41.4%), respectively (Fig. 1).

The cumulative incidence of VTE among patients with *ALK*-negative NSCLC (95% CI) at index and 6-, 12-, 24-, and 60 months post-index were 2.1% (1.7%-2.5%), 7.7% (7.1%-8.4%), 9.9% (9.1%-10.9%), 12.4% (11.5%-13.3%), and 15.1% (14.2%-16.1%) (Fig. 1).

The multivariate cox proportional hazards regression model (Table 2) demonstrated a higher risk for VTE in *ALK*-positive NSCLC as compared with *ALK*-negative NSCLC (HR 1.87 [95% CI, 1.31-2.68]). Past/current smoking was associated with a decreased risk of VTE (HR 0.69 [95% CI, 0.56-0.85]).

**Table 2.** Association of confounding variables with VTE or ATE^*†^.

Variable (at NSCLC diagnosis)	VTE	ATE
	HR (95% CI) ^†^	HR (95% CI) ^†^
*ALK* positive^§^	1.87 (1.30-2.68)	1.24 (0.62-2.47)
Age (increasing)^¶^	0.98 (0.97-0.99)	1.01 (0.99-1.02)
Sex (male)	0.86 (0.71-1.04)	1.14 (0.86-1.53)
Smoker^‡^	0.69 (0.56-0.85)	0.88 (0.65-1.20)
Charlson comorbidity index	0.99 (0.95-1.03)	0.99 (0.93-1.05)
Khorana score	0.96 (0.83-1.10)	0.94 (0.79-1.13)

^*^At any point from 6 months prior study index to 5 years post-index. Index date defined as date of NSCLC diagnosis.

^†^Multivariate cox regression analysis with death as a competing risk.

^§^Defined as ≥2 *ALK*-inhibitor prescriptions (crizotinib or alectinib).

^¶^Hazard ratio for every 1-year increase.

^‡^Past or current smoking.

Abbreviations: *ALK*, anaplastic lymphoma kinase; ATE, arterial thromboembolism; CI, confidence interval; HR, hazard ratio; NSCLC, non-small cell lung cancer; VTE, venous thromboembolism.

### ATE According to *ALK* status

Patients with *ALK*-positive NSCLC had a numerically higher rate of ATE (12/155, 7.7%) compared to *ALK*-negative cases (299/4607, 6.5%); however, *ALK*-positive NSCLC was not significantly associated with a higher risk for ATE (HR 1.24 [0.62-2.47]) (Table 2).

**Figure 2.**
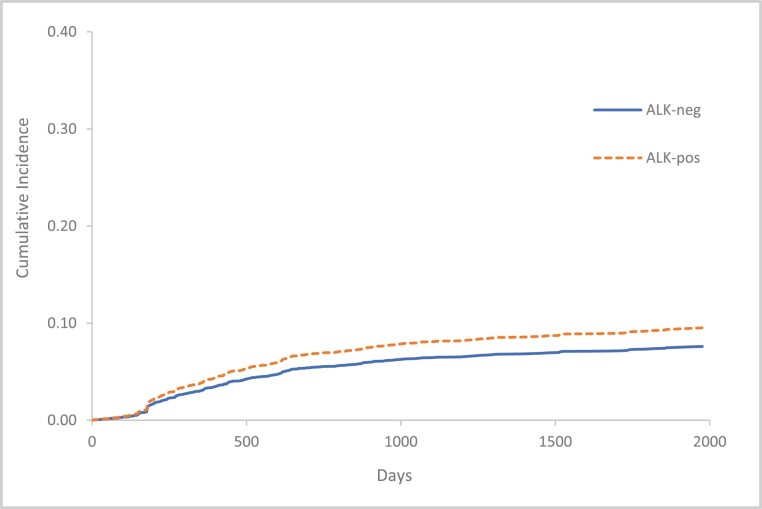
Cumulative incidence of ATE, according to *ALK* rearrangement status. Outcome events counted from 6 months prior study index to 5 years post-index. Index date defined as date of NSCLC diagnosis. Death considered as a competing risk. *ALK*, anaplastic lymphoma kinase; ATE, arterial thromboembolism.

The cumulative incidence of ATE among *ALK*-positive patients (95% CI) at index (representing the prior 6 months) and 6-, 12-, 24-, and 60-months post-index were 1.8% (0.9%-3.3%), 3.93% (2.2%-6.8%), 5.62% (3.1%-10.2%), 7.5% (4.7%-12.7%), 9.52% (5.3%-17.2%), respectively (Fig. 2).

The cumulative incidence of ATE among *ALK*-negative patients (95% CI) at index and 6-, 12-, 24-, and 60-months post-index were 1.4% (1.2%-1.7%), 3.1% (2.6%-3.7%), 4.5% (3.9%-5.1%), 6% (5.3%-6.7%), 7.6% (6.8%-8.5%) as shown in Fig. 2.

## Discussion

This large population-based cohort study demonstrated a higher risk for VTE in *ALK*-positive NSCLC as compared with *ALK*-negative NSCLC. However, *ALK*-positive NSCLC was not significantly associated with a higher risk for ATE.

### VTE in *ALK*-Positive NSCLC

In our study, *ALK*-positive patients had a 6-month VTE incidence of 17.7%, which is similar to prior studies which demonstrated 6-month incidence of 15.7%-22.8%.^18,23^ In contrast to prior studies, the current study includes unselected NSCLC patients treated nationwide at both community-based and tertiary medical centers, improving the generalizability of these findings. The high VTE incidence is comparable to VTE rates seen in patients with high VTE risk malignancies, such as pancreatic and gastric cancer,^24,25^ who are candidates for thromboprophylaxis with direct oral anticoagulants or low molecular weight heparin.^26,27^ In comparison, the VTE rate in unselected NSCLC patients ranges from 3% to 15% in historical studies.^4,7^ Of note, in our study, the Khorana score was not associated with VTE incidence similar to some prior studies of NCSLC patients.^8-10^ Increasing age and past/current smoking were both associated with decreased VTE incidence at 5 years, which may be reconciled by the association between these factors and absence of *ALK*-rearrangement, even after multivariable analysis.

The significant association between the presence of *ALK* alteration and VTE has been reported in several recent studies. In a systemic review and meta-analysis which included 8 trials that slightly vary in their methodology, Zhu et al.^28^ examined thromboembolic events among *ALK/ROS1*-positive patients and reported an odds ratio of 2.1 for VTE in patients with *ALK*-positive NSCLC, in line with our study findings. The high VTE incidence among *ALK*-positive patients with lung cancer may be hypothetically explained by the histopathological features of these cancer cells, which have a mucinous cribriform pattern.^29,30^ Mucin production has been associated with thromboembolism in a murine model,^31^ though evidence of this in patients with lung cancer is lacking.

Taken together, these findings confirm *ALK*-rearrangement as a strong risk factor for VTE in NSCLC and suggest the need for studies investigating thromboprophylaxis strategies in patients with *ALK-*positive NSCLC.

### ATE in *ALK*-Positive NSCLC

Our study also examined ATE incidence, demonstrating that *ALK*-positive NSCLC was not significantly associated with a higher risk for ATE. Data regarding ATE incidence and risk factors among patients with lung cancer, especially in specific molecular subtypes, are scarce.

The association between *ALK*-rearrangement and ATE incidence was previously explored in a single-centre retrospective cohort that demonstrated a non-significant difference of 5% vs. 4.4% (*P* = .71) in the crude ATE rates, though a multivariate regression accounting for cardiovascular risk factors revealed a significantly higher ATE risk attributed to *ALK* genomic alterations (HR 3.15 [95% CI, 1.18-8.37]).^23^ In our study, the crude ATE rate among *ALK*-positive patients was 7.7%, comparable to those in Al-Samkari’s study. Multivariate analysis in the current study did not identify factors associated with ATE, but while age, sex, CCI, and smoking status were available, we did not have available data on specific cardiovascular risk factors such as hypertension and diabetes. These factors are more prevalent in *ALK*-negative lung cancer, as these patients are usually older than *ALK*-positive patients,^15^ possibly explaining why the multivariate analysis did not reveal an association between *ALK* rearrangement and ATE in our study.

Taken together, our study suggests that more research is warranted on ATE risk associated with *ALK*-positive NSCLC, while accounting for multiple ATE risk factors to further test the hypothesis that *ALK-*positive NSCLC might be associated with an increased ATE risk.

## Strengths and Limitations

Strengths of the current study include a dataset from the largest healthcare provider in Israel, which includes all NSCLC patients treated between 2012 and 2019, across a variety of cancer centers nationwide, representing approximately 50% of Israeli population. Our study includes a long follow-up period starting 6 months prior to diagnosis and 5 years after.

Our study has several limitations, some of which are inherent to its retrospective design. First, the use of diagnostic and prescription codes (for VTEs/ATEs and anti-*ALK* tyrosine kinase inhibitors) to classify *ALK* status, rather than manual chart review, introduces possible misclassification bias. For instance, patients with only one anti-*ALK* TKI prescription were classified as *ALK*-negative, although a subset of such patients could potentially have *ALK*-positive NSCLC. This surrogate of *ALK* rearrangement may also lead to misclassification of *ROS1* fusion (also associated with an increased VTE risk^19^) positive NSCLC as *ALK-*positive in a minority of patients (~15% of those classified as *ALK*-positive patients treated after January 2017, when this indication was approved^32^). Second, the registration quality of diagnosis and thromboembolic events may vary between different institutions and family physicians which may affect event rates and risk factor distribution. Third, the *ALK*-positive cohort was probably enriched with patients with adenocarcinoma, as this mutation is nearly exclusively found in patients with this histology.^15,33^ The *ALK*-negative cohort, on the other hand, probably has heterogeneous histologic distribution (where about half are adenocarcinoma and a third are squamous cell carcinoma). Some trials suggest adenocarcinoma in itself is a risk factor for VTE,^34^ while others did not demonstrate such an association.^35,36^ Therefore, this difference in histology may have led to overestimation of the VTE risk associated with *ALK* mutations in lung cancer.

Fourth, the VTE and ATE codes may have been misclassified. These codes have been used in prior studies of patients with and without cancer^37-40^ but have not been formally validated. Another possible limitation is that patients with *ALK*-positive NSCLC have a better prognosis and, supposedly, “more time” to develop thromboembolic events. Nevertheless, this potential confounder was addressed by adjusting for death as a competing risk in analyses with VTE/ATE as the dependent variable, and by treating VTE and ATE as time-dependent variables in analyses with death as the dependent variable. Last, residual confounding is possible since we did not evaluate all potential risk factors for ATE and VTE.

## Conclusion

*ALK*-positive NSCLC was associated with VTE but not ATE. VTE incidence in patients with *ALK*-rearrangement may be sufficiently high to consider thromboprophylaxis, but this needs to be assessed in future studies.

## Supplementary Material

oyad061_suppl_Supplementary_Material

## Data Availability

The data underlying this article were provided by Clalit Health Services under licence, by permission. Data will be shared on request to the corresponding author with permission of Clalit Health Services.

## Previous Presentation

This study was previously presented at the 2019 ASCO Annual Meeting.
