# Supplementary material for: Arterial and Venous Thromboembolism in ALK-Rearrangement-Positive Non-small Cell Lung Cancer: A Population-Based Cohort Study
Source: Oncologist. 2023 Apr 4;28(6):e391–6. doi: 10.1093/oncolo/oyad061 (PMC10243788; doi:10.1093/oncolo/oyad061)
Supplement: oyad061_suppl_Supplementary_Material [file oyad061_suppl_supplementary_material.docx]

**Arterial and venous thromboembolism in *ALK*-rearrangement positive non-small cell lung cancer: a population-based cohort study**

**ONLINE-ONLY APPENDIX**

**Content:**

1. **Online table 1:** ICD-9 codes used for deep vein thrombosis, pulmonary Pg. 2
   embolism and arterial thromboembolism.
2. **Supplemental results:** Overall VTE and ATE incidence Pg. 3
3. **Supplemental Figure 1:** Cumulative incidence of VTE and ATE in all patients Pg. 4

**1) Supplemental Table 1**: ICD-9 codes used for deep vein thrombosis, pulmonary embolism and arterial thromboembolism.

**2) Supplemental results:** Overall VTE and ATE incidence

***VTE incidence***

Overall, 673 (14%) patients had a VTE during follow up. The cumulative incidence of VTE (95% CI) with death as a competing risk, at index (representing the prior 6 months), and 6-, 12-, 24-, and 60-months post-index were 2.2% (1.8-2.6%), 8% (7.3-8.8%), 10.42% (9.4-11.5%), 13% (11.9-14%), and 15.7% (14.7-16.6%), respectively, as shown in figure 1A.

**ATE incidence**

Overall, 311 (6.5%) of patients experienced an ATE during follow up. The cumulative incidence of ATE (95% CI) at index (representing the prior 6 months) and 6-, 12-, 24-, and 60-months post-index were 1.4% (1.2-1.7%), 3.15% (2.6-3.8%), 4.5% (3.9-5.1%), 6% (5.5-6.7%) and 7.6% (6.8-8.6%), respectively, as shown in figure 1B.

**3) Supplemental Figure 1:** Cumulative incidence of VTE and ATE in all patients

**A**

**B**

This figure shows the cumulative incidence of VTE (Panel A) and ATE (Panel B) in all patients. Outcome events were counted from 6 months prior study index to 5 years post-index. Index date defined as date of non-small cell lung cancer diagnosis. Death considered as a competing risk.

*ATE*, arterial thromboembolism; *VTE*, venous thromboembolism
